# Progress and emerging techniques for biomaterial-based derivation of mesenchymal stem cells (MSCs) from pluripotent stem cells (PSCs)

**DOI:** 10.1186/s40824-023-00371-0

**Published:** 2023-04-18

**Authors:** Nityanand Prakash, Jiseong Kim, Jieun Jeon, Siyeon Kim, Yoshie Arai, Alvin Bacero Bello, Hansoo Park, Soo-Hong Lee

**Affiliations:** 1 Department of Biomedical Engineering, Dongguk University, Seoul, 04620 Korea; 2 School of Integrative Engineering, Chung-Ang University, Seoul, 06911 Korea

**Keywords:** Mesenchymal stem cell (MSC), Induced pluripotent stem cell (iPSC), iPSC-derived MSC, MSC derivation, Biomaterial

## Abstract

The use of mesenchymal stem cells (MSCs) for clinical purposes has skyrocketed in the past decade. Their multilineage differentiation potentials and immunomodulatory properties have facilitated the discovery of therapies for various illnesses. MSCs can be isolated from infant and adult tissue sources, which means they are easily available. However, this raises concerns because of the heterogeneity among the various MSC sources, which limits their effective use. Variabilities arise from donor- and tissue-specific differences, such as age, sex, and tissue source. Moreover, adult-sourced MSCs have limited proliferation potentials, which hinders their long-term therapeutic efficacy. These limitations of adult MSCs have prompted researchers to develop a new method for generating MSCs. Pluripotent stem cells (PSCs), such as embryonic stem cells and induced PSCs (iPSCs), can differentiate into various types of cells. Herein, a thorough review of the characteristics, functions, and clinical importance of MSCs is presented. The existing sources of MSCs, including adult- and infant-based sources, are compared. The most recent techniques for deriving MSCs from iPSCs, with a focus on biomaterial-assisted methods in both two- and three-dimensional culture systems, are listed and elaborated. Finally, several opportunities to develop improved methods for efficiently producing MSCs with the aim of advancing their various clinical applications are described.

**Supplementary Information:**

The online version contains supplementary material available at 10.1186/s40824-023-00371-0.

## Introduction

The use of stem cells for cell therapy has recently increased. This is especially true for adult stem cells, which are used to treat various injuries. Mesenchymal stem cells (MSCs) are a type of adult stem cells that have shown promising potential in clinical trials for treating various diseases [1, 2]. The therapeutic properties and large-scale use of MSCs have been researched extensively, and different methods have been developed for their isolation and production. MSCs are used owing to their self-renewal capabilities, multipotency, paracrine effects, immunoregulatory, and other cell-supportive properties [3, 4]. They regulate their own survival and impact the growth of neighboring cells or cells in the transplanted site. Their differential potential has been studied comprehensively and explored for the production of osteoblasts, adipocytes, chondrocytes, neurons, and myocytes [5]. These fibroblast-plastic-adherent cells are identified and isolated based on the presence of cluster of differentiation (CD) markers, including CD73, CD90, CD105, and stromal precursor antigen-1 (Stro-1) markers, and the absence of CD34, CD45, CD19, CD11, and human leukocyte antigen (HLA)-DR markers. This surface marker characterization was established by the International Society of Cellular Therapy (ISCT) to separate MSCs from heterogeneous populations of mononuclear adherent cells [6]. In addition to their multipotency, MSC sources are present throughout the body with little variation in their differentiation potential and surface markers across locations, and all of them can be used for cell therapy [5].

Generally, there are two mechanisms underlying the action of MSCs in treating diseased or damaged tissues or cells. The first is attributed to their multilineage differentiation capacity. They home to and engraft within injured sites and then differentiate into end-stage functional cells, thereby replacing the injured cells at those sites. Many articular cartilage injuries, for instance, injured knees, are treated by introducing MSCs into the damaged tissue, which then differentiate into articular chondrocytes to replace the damaged cartilage [7]. The second is the paracrine mechanism of MSCs. It has been well established that MSCs release various cytokines, such as angiogenic and neurogenic factors and immunomodulatory cytokines [8–10]. These factors promote the repair and regeneration of damaged tissue. Although MSCs have been widely used for clinical cell therapy, they have few limitations. For instance, MSCs have a limited self-renewal capacity when cultured in vitro. In addition, studies have demonstrated that they tend to lose their regenerative properties when cultured for long periods [11]. Furthermore, their source must be managed properly and subjected to quality control to avoid batch-to-batch variations. With the current developments in induced pluripotent stem cell (iPSC) technology, iPSC-derived MSCs can eliminate the drawbacks associated with the MSCs derived from the existing sources.

This review examines the molecular morphology and medical applications of MSCs in treating various diseases and injuries. Here, the existing sources of adult MSCs are compared, with a focus on their isolation methods, proliferation rate differences, and multilineage differentiation potentials. Moreover, limitations in the clinical use of adult MSCs and approaches to overcome these drawbacks by generating MSCs from iPSCs are discussed. Finally, the existing methods for the differentiation of iPSCs into clinically relevant MSCs are identified and described. Moreover, recent advancements in two-dimensional (2D) and three-dimensional (3D) biomaterial-based directed differentiation of iPSCs into MSCs and the effects of various inducing growth factors are listed and compared.

## MSCs: characteristics and potential promise for cell and tissue engineering

With changing lifestyles, health issues are increasing, and new drugs are being developed to treat these issues. However, therapeutic drugs often have serious side effects and low success rates. Cell therapy is an alternative and promising treatment approach. Cell therapy involves the use of cells instead of chemicals, and it often yields similar results in vitro and in vivo. For instance, when MSCs are used therapeutically at a bone injury site, they can expand, differentiate into osteoblasts, and release essential growth factors and paracrine molecules that support osteoblast proliferation [12]. Human embryonic stem cells (hESCs), iPSCs, and MSCs are among the most suitable sources of stem cells for use in cell therapy. It is important to note that the use of ESC is still debatable due to the possibility of teratoma formation and the ethical concerns regarding their isolation (destruction of an embryo) and thus are less preferred compared with iPSCs [13]. By contrast, MSCs are proven safe and have been used in several clinical trials [14, 15]. They are multipotent cells that can self-regenerate and can be grown in vitro. MSCs possess fibroblast-like morphology with plastic-adherent ability, which allows them to grow in tissue culture plates without requiring additional substrates.

### Cellular and molecular properties of MSCs

MSC-like cells were first discovered by Friedenstein in 1970 in guinea-pig bone marrow (BM) [16]. However multiple cell types in the BM led to controversy in selecting a specific name for these cells. Later, the ISCT termed these cells multipotent mesenchymal *stromal* cells based on specific properties such as having a fibroblast morphology, and plastic-adherence capability [17].

Later, many surface markers specific to these cells were found; the CD on their surface allowed for the identification of MSCs from other groups of plastic-adherent cells. The presence of CD73, CD90, CD105, and Stro-1 and the absence of CD45, CD34, CD14, CD19, and HLA class II identified the presence of MSCs in cell cultures [18–22]. These characteristic markers are used for their isolation as well.

Slight differences in the expression of surface markers may occur based on the origin of MSCs. Depending on the source of MSC (adipose tissue, bone marrow, umbilical cord), some MSC populations have different cell surface markers, which may contribute to and be reflected in their differentiation potentials [5, 23–25]. These differences allow the selection of a particular type of MSCs for a specific therapeutic purpose.

MSCs are multipotent cells that can differentiate into different types of cells. Generally, they are recognized for their potential to differentiate into osteoblasts, chondrocytes, and adipocytes. However, they not only differentiate into mesoderm-type cells but also into other lineages, such as cardiomyocytes, neurons, germ cells, and hepatocytes, and this is achieved by altering various signaling pathways during the development of MSCs to induce specific differentiations [26–28]. Treating different growth factors can alter the differentiation potential of MSCs [26, 28, 29].

Another important property of MSCs is their ability to dodge the immune system and improve transplantation efficiency. The major histocompatibility complexes MHC-I and MHC-II are proteins that play a major role in the recognition of allogenic cells by the host immune system. Low expression of these molecules in MSCs enables them to be not recognized by the host’s immune system [8, 30, 31]. In addition, the immunomodulation capability of MSCs promotes angiogenesis, inhibits apoptosis and inflammation, and modulates the extracellular matrix (ECM) through paracrine factors [32]. They are also involved in suppressing the proliferation of T cells and the maturation of dendritic cells (DCs) and monocytes [33]. Overall, MSCs can control the immune regulatory system of the body by promoting or suppressing proteins that have important functions in the immune system.

**Fig. 1:**
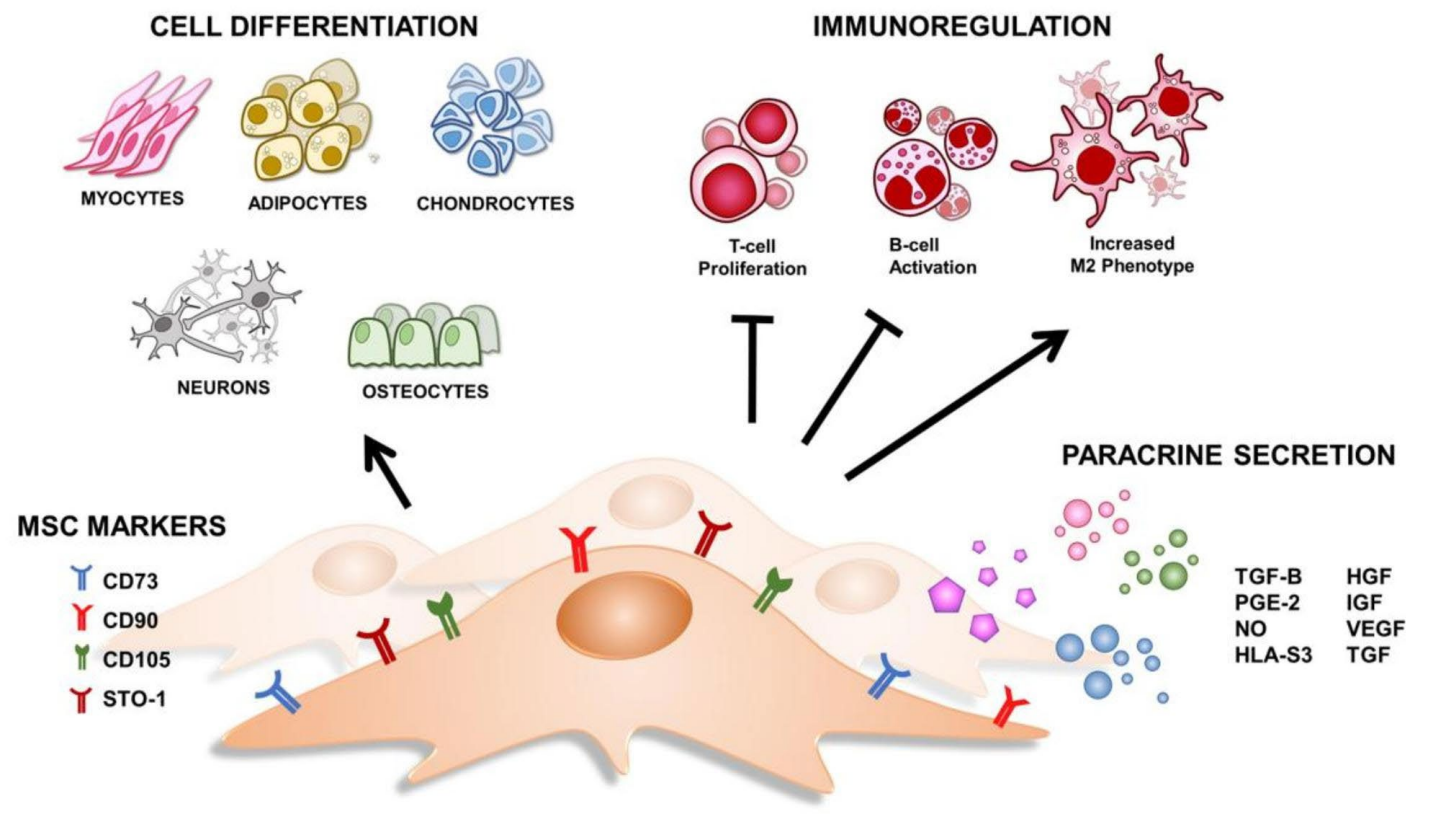
**Cellular and molecular properties of MSCs.** MSCs display multipotency and different CD markers. They are great candidates for immune-regulation by means of T-cell and B-cell inhibition, as well as M2 phenotype encouragement. Paracrine secretion of different growth-regulating and inhibitory factors provides them distinct therapeutic properties to support other cells

The paracrine effect of MSCs additionally plays a major role in injury treatment and cell growth. MSCs release around 43 angiogenic factors, among which 11 factors are involved in bone regeneration; 2 in neuroprotection; and the other 30 in vascular repair, peripheral artery disease, and myocardial infarction treatment [9, 10, 34]. Moreover, immunomodulatory effects are managed by paracrine factors. making release of cytokines and growth factors has been observed to inhibit immune cell proliferation [35]. Overall, these results demonstrate that the properties of MSCs can significantly affect healing and regeneration. A diagram depicting all these characteristics is presented in Fig. 1.

### Clinical applications of MSCs

Owing to the low efficacy and efficiency of drugs available for treating several diseases, a shift has occurred toward cell therapy, and recognition of the therapeutic potential of MSCs is perhaps the most exciting advance in cell therapy [36–38]. Recently, MSCs have been profusely used in cell replacement strategies to treat cartilage injuries caused by chronic load, autoimmune diseases such as arthritis, and physical trauma. For instance, autologous transplantation of BM-derived MSCs embedded in a 2-mm-thick collagen gel onto a patient’s patella led to improvement in the patient’s movement and promoted complete regeneration of the cartilage tissue [39–41]. MSCs derived from other sources have yielded similar results, thereby proving the benefits of MSCs in treating osteoarthritis and other cartilage-associated defects [7, 40–47].

Furthermore, MSCs have been used to treat various bone defects caused by bone loss, fragility, and fracture. With technological advancements, various biomaterials have been used in combination with MSCs to achieve improved results. MSCs were successfully combined with a bovine bone mineral to reconstruct the atrophic maxilla [48]; this approach facilitated bone formation in the elevated region owing to the presence of MSCs in the biomineral. Similarly, MSCs combined with bio-ceramics have been used to treat long bone non-unions [49].

Other clinical applications of MSCs include treatment of spinal cord injuries, graft-versus-host diseases (GVHDs), and various cardiovascular diseases [50–54]. Moreover, the injection of autologous BMMSCs at injury sites led to improvements in motor power, daily motor tasks, and recovery of injured areas. These improvements, however, were subjective and patient-dependent [50, 51].

**Fig. 2:**
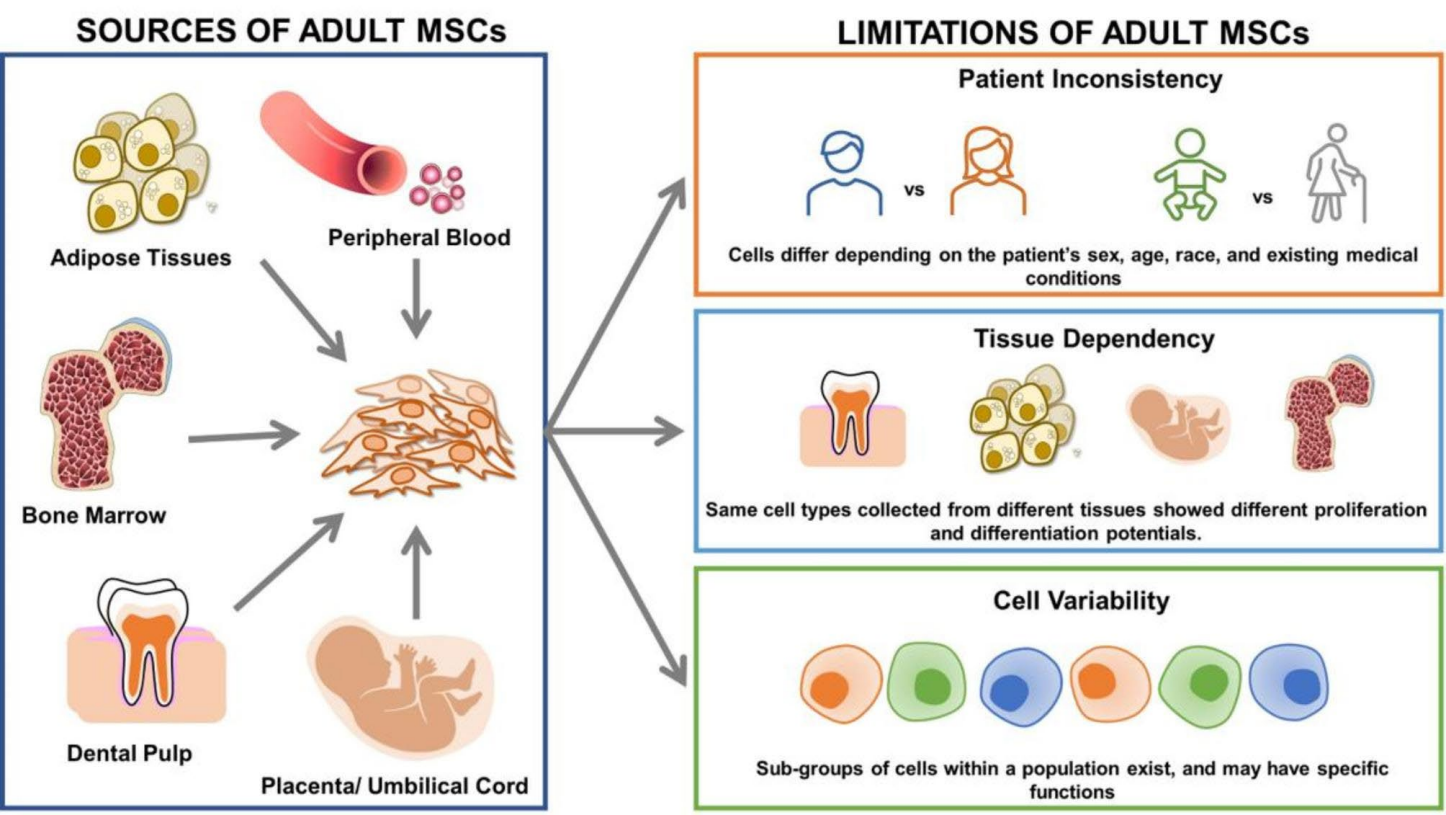
**Sources and heterogeneities of MSCs.** Heterogeneity in MSCs can be classified into different levels: donor-to-donor inconsistencies, tissue-dependent variations, and the presence of sub-populations. Due to these variations, the therapeutic potentials of MSC are hindered, which encouraged scientists to shift their focus towards the development of a new source of MSCs.

Although the use of MSCs for cell therapy has paved ways in treating various diseases, there are still some limitations. First, adult MSC have limited proliferation capacity. Thus, a constant supply of adult MSCs is needed to sustain treatment. Although this can be solved by obtaining MSCs from several donors, cells obtained from various donors and tissues have differences. In general, MSC condition depend on the donor’s sex, age, race, and existing pre-medical conditions (Fig. 2) [55, 56]. A detailed explanation of the limitations of MSCs is discussed in the following sections.

### Current sources of MSCs and their limitations

It has been well established that MSCs derived from various tissues and donor sources have different proliferation and differentiation potentials. Therefore, it is imperative to know the source of MSCs to establish whether they are fit for purpose and ensure a successful outcome. These sources can be categorized into two types based on the age of the donor: adult and infant sources. Adult tissue sources include BM, AD, dental pulp, peripheral blood, synovium and synovial fluid (SF), endometrium, skin, and muscle. Infant tissue sources include amniotic fluid, the placenta, umbilical cord blood (UCB), and Wharton’s jelly (WJ) [5, 57].

In general, adult-sourced MSC are easily accessible but with lower proliferation capacity. On the other hand, infant-sourced MSCs have higher proliferation but are not always available [14]. Specific differences and limitations are discussed in the subsequent sections.

#### Adult tissue sources

The major sources of adult MSC in the human body are bone marrow and adipose. BM was considered the only multipotent MSC source after its discovery in 1970 [58]. Isolation of MSCs from BM is rather invasive and painful, but cells isolated from BM can differentiate into adipocytes, chondrocytes, osteocytes, cardiomyocytes, neurons, and many other types of cells [5, 59]. The immunoregulatory effects of these cells are weaker than those of umbilical cord-derived MSCs (UCMSCs), but their immunomodulatory properties are adequate, and they promote angiogenesis, myogenesis, and growth and development of other cells, which make them a great candidate for cell therapeutics [8, 60, 61].

Another source of MSCs is AD tissue, which is easily accessible and abundant throughout the body. Adipose-derived MSCs (ADMSCs) are isolated from the subcutaneous AD tissue harvested by liposuction or as large pieces of tissue [62]. They exhibit high plasticity, which leads to differentiation into various cell types, such as adipocytes, chondrocytes, osteoblasts, cardiomyocytes, neuronal cells, hepatocytes, and, more recently, germ cells; moreover, they have immunosuppressive effects and release more angiogenic regulators [5, 63–65].

Dental pulp-derived MSCs (DPMSCs) are isolated from the dental pulp tissue of the third molar teeth, deciduous teeth, or any extracted teeth [66, 67]. They are easier to isolate, have a high proliferative rate, and provide adequate amounts of MSCs for therapeutic use, despite the small area of teeth. Nonetheless, their differentiation potency is limited to neural ectoderms, myoblasts, osteoblasts, chondrocytes, adipocytes, and odontoblasts, compared to that of bone marrow-derived mesenchymal stem cells (BMMSCs), which have vast plasticity [67]. Other sources of MSCs include synovium, SF, and synovial membrane [68–70].

#### Infant tissue sources

Similar to adult tissue sources, MSCs can be isolated from various infant tissues. These sources of MSCs are available starting from embryo development until the infant’s birth, and they include tissues from the embryo and the mother [25].

The umbilical cord (UC) is a tissue composed of the umbilical arteries (UCAs) and umbilical veins (UCVs) and is surrounded by a gelatinous extracellular membrane called WJ [71]. UCs are considered medical waste and are, therefore, an easily and noninvasively available source of MSCs. MSCs isolated from UC arteries and veins are often called UCMSCs while the ones isolated from the WJ are called WJMSCs. It is important to note, however, that these terms are used interchangeably in most publications. These UCMSCs/WJMSCs have a high proliferative rate, short doubling time, are abundant, and exhibit excellent immunosuppressive properties, and were found to prefer chondrogenic differentiation [31, 72–78].

Umbilical cord blood (UCB) is another source and termed as UCBMSC that is abundantly available and easily retrieved [79, 80]. These MSCs have high doubling number, can be cultured for higher passages and can efficiently differentiate into osteocytes and chondrocytes compared to BMMSC and ADMSC. In some studies, it is suggested that they outperform WJ-MSCs by releasing anti-fibrotic factor HGF and genes associated with ECM remodeling, which are helpful in scarless wound healing [79–81]. Likewise, placenta and amniotic fluid demonstrated MSC like properties with good immunogenic properties and high proliferative rate [82–84].

#### Limitations and heterogeneity of existing MSC sources

**Fig. 3:**
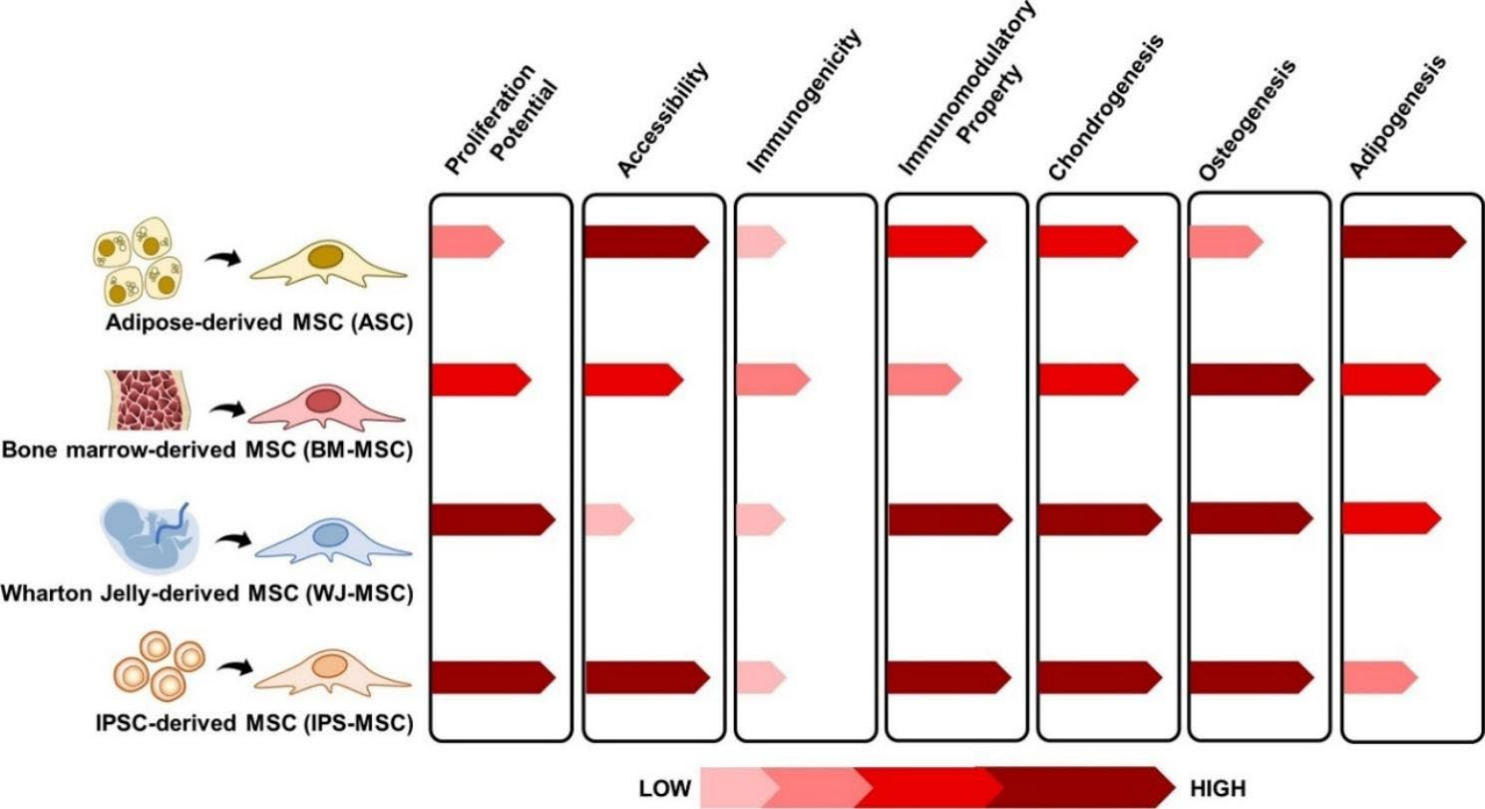
**Comparative analyses between several types of MSCs.** MSC have multiple sources of origin such as bone marrow, adipose tissue, umbilical cord, dental pulp, and peripheral blood. Each source has varying level of accessibility and isolation method. Moreover, depending on the source of MSC, differences in proliferation capacity, differentiation potentials, immunogenicity, and immunoregulation are observed. Comparison was based from the most current studies on MSC.

Although most types of MSC have same cellular morphology and properties, these cells are also found to have differences in molecular phenotype and thus functions. The heterogeneity among various MSC populations can be subdivided into three categories: Donor-to-donor inconsistencies, tissue-dependent variations, and existence of cell sub-populations. The heterogeneity of MSCs is depicted in Figs. 2 and 3.

The first level of heterogeneity in MSC is the donor-to-donor variations. The age, sex/gender, and the presence of pre-existing medical conditions of the donors are found to affect MSC proliferation, differentiation, and therapeutic potentials. For instance, BMMSCs derived from a female donor showed higher therapeutic efficacy compared to BMMSC from a male donor [56]. Similarly, MSCs isolated from young donors have higher proliferation, increased anti-inflammatory cytokine secretion, and enhanced immunoregulation compared to MSCs from an old donor [85]. Finally, the donor’s pre-existing medical conditions and genetic variations are other types of limitations that affect MSC properties [11, 86].

The next level of heterogeneity is the tissue-specific differences of various MSCs. Depending on the tissue source, isolation procedure, accessibility, and differentiation potentials may vary. BMMSCs have been used in clinical trials for osteogenic and chondrogenic injuries, as well as cardiovascular and neurogenic diseases, among others. BMMSCs have great potential, but their retrieval procedure causes pain, potentially life-threatening infection, and donor site morbidity in patients [87]. In addition, the quantity of harvested cells is limited. MSC collection from the BM isolates only approximately 0.001−0.01% of the total MSCs, which need to be expanded in vitro to a higher passage number. Consequently, the isolated cells lose their potency and genetic stability [65, 88, 89]. In terms of differentiation potentials, BMMSCs have better chondrogenic and osteogenic potentials than adipogenesis.

On the other hand, adipose tissues (AD) provide tenfold more MSCs than bone marrow. These tissues are more accessible, and the tissue collection is less invasive compared with the bone marrow [24]. Having an adipose origin, ADMSCs prefer adipogenesis but are also effective in capable of osteogenesis and chondrogenesis. In contrast, UCMSCs involves a more noninvasive isolation method and provide an abundant supply of stem cells, but they are not always available timeously because they can be obtained only when an infant is born, which limits their supply [75]. Compared with other sources, UCMSCs can efficiently undergo chondrogenesis, osteogenesis, and adipogenesis.

The final level of heterogeneity involves the presence of several sub-populations of MSCs.

For instance, the CD105^−^ ADMSCs exhibit stronger osteogenic potential than the CD105^+^ cells [90–93]In a parallel study, CD271^+^ BMMSCs are superior in repairing articular cartilage defects [94, 95]. On the other hand, CD200^+^ BMMSCs have shown better osteogenic and immunoregulatory effects than CD200^−^ MSCs [96]. A list of various sub-populations of MSCS are listed in Table 1.

**Table 1:** **Different existing sub-populations of MSC.** Various sub-populations exist within a particular type of MSCs. Based on the presence of cell surface markers, some sub-population may have different properties, and thus may be used for a specific function

MSC subpopulation	Source	Differentiation capacity and Enhanced Properties	References
***CD105+***	AD and UC	Enhanced angiogenesis, chondrogenesis, and increased proliferation	(97)
***CD105-***	AD and UC	Enhanced osteogenesis, higher immunosuppressive ability	(91,98)
***CD200+***	BM and UC	Enhanced osteogenesis and immunoregulation	(92)
***STRO-1+***	BM, DP, SM	Arteriogenesis, cardiac recovery and homing ability	(88)
***CD271+***	AD, BM and synovium	High CFU, enhanced HSC engraftment, superior chondral repair, high proliferation	(90,91)

These underlying heterogeneities of MSC populations hinders its therapeutic potentials. Although adult- and infant-sourced MSCs have been widely used in cell therapies, they are insufficient as there is still a need for a constant supply of healthy, highly proliferative, and homogenous population of MSCs for medical purposes. Thus, there is a high demand to differentiate PSCs into functional MSCs. For instance, in recent years, MSCs have been derived from iPSCs, and they are called induced mesenchymal stem cells (iMSCs). iMSCs have reformed the MSCs used in therapeutics because they overcome the limitations of conventional MSC sources. Based on the most recent studies in MSC, a comparison between BMMSC, ASC, UCMSC, and PSC-derived MSC is shown in Fig. 3.

## Biochemical and physiochemical factors affecting MSC fate

To better understand MSC development, it is important to understand the micro-environment or niche in which these cells reside in-vivo. Several factors associated with these niches, such as biochemical and physiochemical cues, regulate molecular processes such as cell proliferation, differentiation, and survival. Understanding these microenvironments in-vivo allows scientists to mimic cell development in-vitro and maximize the use of cells, in this case MSCs, for therapeutic purposes. Because MSCs have multiple tissue sources, we focused on discussing the bone marrow microenvironment where BMMSCs are located. The BM microenvironment is a dynamic region composed of several cell types, growth factors, and cytokines. It is a rich area that supports the production and development of all blood cells in the body. In this section, we chose to discuss BMMSCs as these cells are one of the longest and most utilized cell sources for stem cell therapy. In comparison with other types of MSCs, BMMSCs have shown better multi-lineage potential than ADMSCs, and are more accessible compared with UCMSCs. Finally, the biochemical and physiochemical cues provided by the BM for BMMSCs proliferation, migration, differentiation, and paracrine secretion have also been well established.

### Biochemical factors affecting MSC development

In bone marrow, MSCs reside and interact with various cells such as osteoblasts, osteoclasts, immune cells, pericytes, and hematopoietic cells. These cells secrete various factors and signaling molecules that affect MSC proliferation, fate decision, paracrine secretion, and differentiation. A complex network of signaling pathways is involved in MSC development, but only a few MSC signaling pathways have been explored thus far. These signaling pathways are strongly influenced by several growth factors, hormones, and cytokines [99, 100]. Well-studied key regulatory factors include growth factors such as TGFβ1, IGF1, bFGF, and VEGF and cytokines such as IL-6, IL-1β, IFN-γ, and TNF-α. In addition to these, oxygen, calcium, and the other hormones that are generally released in diseased conditions play an important role in MSC development [101, 102].

In the BM, numerous studies suggest that MSCs are in quiescence (G0) state in the perivascular spaces. When the body perceives any local or distant injury or tissue damage, they become activated, are induced to proliferate, and are the signaled to migrate to injury sites to promote healing and tissue repair [103]. Depending on the location in which MSCs are needed, the MSCs are then induced to differentiate into a specific cell type, i.e. osteoblasts for bone repair or chondrocytes for cartilage repair. The ability of the MSCs to self-proliferate or also called “stemness” is regulated by cytokines and growth factors such as IL-6 the TGFβ proteins, BMPs, EGF, PDGF, and others [104]. Activated and proliferated cells then migrate and home to the damaged tissue via the upregulation of Stro-1 surface proteins [105]. Here they differentiate in response to the upregulated cytokines or growth factors in the injury site. In bone fractures, for instance, macrophages in the injury site secretes IL-1, IL-6, and tumor necrosis factor-alpha (TNF-α) that recruits MSC to start the process of healing and osteoblast differentiation [106]. Other growth factors of MSCs proliferation and differentiation are discussed below.

One of the most abundant growth factors present in bone matrix is IGF-1, and has been determined to be involved in the proliferation, self-renewal, and in some parts, osteogenic differentiation of MSCs [107–109]. IGF-1 is primarily involved in proliferation, but during the process of MSC differentiation, it additionally helps with the mineralization of cells through the activation of the mTOR pathway, indicating its possible dual role in MSC regulation [107, 110]. In a parallel study, the presence of IGF-1 promoted the enhanced proliferation of MSCs under hypoxic conditions [111].

Another abundantly available growth factor (GF) in the BM microenvironment is the TGFβ family of proteins composed of TGFβ1, TGFβ2, and TGFβ3, and all BMPs. It is well established that the TGFβ family regulates MSC development. For instance, TGFβ1 binds to MSCs during bone remodeling, induces the migration of MSCs to the target location, and promotes the chondrogenic and osteogenic differentiations of MSCs [99, 112, 113]. Another protein belonging to the same family is BMP4, which is primarily involved in adipocyte generation [114]. The TGF family has additionally been found to regulate MSC proliferation through crosstalk with the Wnt signaling pathways [115]. A study demonstrated that TGFβ1 supports Wnt signaling through the activation of Smad3, which induces enhanced nuclear translocation of β-catenin, and thus, increased proliferation. Interestingly, this crosstalk reduces the osteogenic differentiation of MSC and is, therefore, important for maintaining the self-renewal capacity of MSCs [116]. Similar crosstalk was observed between TGFβ1 and FGF2, which promotes MSC proliferation, whereas HGF and IGF-1 promote osteogenesis [117].

Other chemical cues present in the bone microenvironment include several cytokines and oxygen. Cytokines such as TNFα, IL-1β, and IL-6 were found to be involved in the differentiation of MSCs into osteoblasts [99]. Oxygen, by contrast, has been found to regulate both the proliferation and differentiation of MSCs. A lower oxygen concentration supports MSC proliferation, while a higher oxygen concentration induces osteogenesis and angiogenesis [111, 118].

### Physiochemical factors affecting MSC development

To develop a suitable biomaterial-based platform for the differentiation of iPSCs into MSCs in vitro, it is important to identify the physiochemical factors that regulate MSC development in-vivo. Herein, factors such as ECM composition, microenvironment stiffness, and topography are discussed. The bone is a spongy microenvironment comprising several minerals (hydroxyapatite, calcium), proteins (ECM proteins, Type I collagen, laminin), lipids, and water [119]. The concentration and arrangement of these molecules ultimately affect bone fragility and help maintain angiogenesis, oxygen regulation, and overall growth of MSCs [120–122].

Collagen is one the most abundant ECM proteins in BM. The presence of collagen not only provides tensile strength but also allows for the mineralization of calcium and other ions and eventually creates a special microenvironment for cell growth and survival, including MSCs. Additionally, collagen allows for enhanced cell adherence and proliferation of MSCs by reducing their doubling time [123]. Other ECM proteins such as fibronectin and laminin, too, regulate the development of MSCs by interacting with the various integrins expressed by MSCs [124, 125]. Aside from ECM proteins, several minerals have been found to determine the fate of MSCs in the BM niche. For instance, calcium ions enhance bone regeneration by promoting MSC migration and inducing osteoblast generation [126]. Such regulated calcium release was achieved in vitro on a hydroxyapatite scaffold to induce osteoblast differentiation of MSCs [127].

BM is composed of the endosteal niche, which is heavily populated with various bone-forming cells, and the vascular niche, which is populated with endothelial cells, adipocytes, and stromal cells [128, 129]. These niches differ in terms of cell composition, stiffness, oxygen concentration, and for this reason, they have varying effects on cell development in BM. MSCs can be localized in both niches, and therefore, it is imperative to note that the properties of these niches affect the general fate of MSCs [130]. The endosteal niche is relatively stiffer (35–40 kPa) than the vascular niche (3 kPa). In vitro studies have attempted to determine the effect of ECM stiffness on MSCs by mimicking its in-vivo condition. It has been demonstrated that the stiffer fibronectin-coated polyacrylamide hydrogel (13–68 kPa) supports BMMSC adhesion and spreading [131]. Moreover, chondrogenic and osteogenic differentiation has been found to be greatly enhanced when BMMSCs are seeded in 4-methyl acrylate polymer, which has a stiffness of 10 MPa [132]. This can be attributed to the fact that in vivo, bone formation usually occurs in the stiffer endosteal niche, where MSCs are recruited and programmed to differentiate into bone-forming cells. Other important properties of this niche that can affect MSC fate include porosity and topography of the ECM. Although only a few studies have been conducted to determine these factors in-vivo, several attempts have been made to determine them in vitro. In Vissers et al.’s study, a scaffold with a pore size of 500–850 μm, exhibited the strongest osteogenic response [133]. In a similar study, a scaffold with cubic pores greatly increased the gene expression of MSCs undergoing chondrogenesis and adipogenesis compared to a scaffold with cylindrical pores [134]. In terms of topography, it is known that cells can detect surface patterns of matter from 10 nm to 100 μm and control their ability to differentiate and proliferate. In one study, a 15-µm ridge increased the efficiency of adipogenic differentiation, while a 2-µm ridge increased the efficiency of osteogenic differentiation [135]. In another study, the MSCs grown on micro-grooved surfaces exhibited higher expressions of pluripotency-related markers and two to threefold faster proliferation capabilities compared to those of the MSCs grown in a conventional culture flask [136].

## Existing strategies for differentiating PSCs into MSCs

Because the sources of MSCs are limited and incapable of retaining their characteristics for long periods in in-vitro environments, a stable source of MSCs is required. Pluripotent stem cells (PSCs) have the potential to overcome these limitations and serve as a superior source of MSCs than the existing sources. This conversion is directed through a particular route. Conventionally, PSCs are first converted into mesodermal cells and then differentiated into MSC-like cells. A few researchers have directly converted PSCs into MSCs. In one study, MSC-like properties were induced in somatic cells by transducing human adult blood CD34^+^ with lentivirus OCT4 under MSC-inducing conditions [137]. The different routes of and recently developed methods for producing MSCs from iPSCs are described below and summarized in Table 2, while the advantages and disadvantages of each technique is summarized in Table 3.

**Table 2:** **Existing techniques for generation of MSCs from PSCs.** Various methods such as growth factor induction, embryonic body formation, gene modification, and biomaterials have been utilized to differentiate various PSCs into MSCs.

Type of Technique	Method	Cells used	MSC properties	Publication
**Growth Factors**	BMP4	hESCs	EMT gene upregulation	(158)
	BMP4 with activin inhibitor (A83-01) in MesenCult™ media	hESCs	3–4 weeks for differentiation; CD73^+^, CD90^+^, and CD105^+^ cells with trilineage properties	(135)
	ESCs were cultured with SB431542	hiPSCs	MSCs were generated rapidly in 10 days, and they exhibited good trilineage potential and efficient passaging capability	(140)
	IκB kinase/NF-κB signaling inhibitor and p65 inhibitor were used with MSC culture medium	hiPSCs	MSCs with high osteogenic and chondrogenic properties	(155)
**Embryoid body**	ESCs were cultured in suspension culture and outgrowths were isolated	hESCs	10-day differentiation; CD73^+^, CD90^+^, and CD105^+^ cells with chondrogenic properties	(147)
	ESCs were cultured in suspension culture. The EBs formed were plated in 0.1% gelatin low-glucose media, and outgrowths were isolated	hESCs	MSCs markers were observed, and the expressions of the CD73 and CD105 markers increased with the passage number	(148)
	Cells were plated in low-attachment plates, and the EBs formed were replated in 0.1 gelatin with MSC media	iPSCs	After 35 days, MSC-like cells were generated with low CD105 expression, immunomodulatory properties, and adipogenic potential	(149)
	EBs were formed in non-tissue culture plates with knockout serum replacement medium, transferred to the MSC medium, and treated with SB431542	iPSCs and hESCs	MSCs were generated in 10 days with SB431542 treatment; the CD105 and adipogenic potential were low. The other markers were normal, osteogenic and chondrocyte potential were normal, and proliferation rate was higher	(139)
	EBs were formed with BMP4, bFGF, and VEGF on a low-attachment surface. Mesangioblast were formed, which were then cultured in serum-free methylcellulose medium over Matrigel®	hESC	The output was higher than that of BMMSCs with higher doubling rate and smaller size; the Stro-1 marker was lacking	(160)
**Other techniques**	OP9 stromal cells were co-cultured with hESC; CD73^+^ cells were isolated with FACS and cultured	hESC	Induction time was longer at 40 days. Other than CD90, all markers were present, and the trilineage potential was good	(150)
	Repeated passage of ESCs on gelatin-coated plates	hESC	Passage pressure generated MP, which exhibited homogeneous population, tumor-free nature, and good tri-lineage potential	(155,161)
	Lentivirus-induced HOX genes in miPSCs	miPSCs	HOX gene was overexpressed, and vascular wall MSCs were generated	(152)
	DOX-induced MSX2 overexpression in hiPSCs in MSCs medium	hiPSCs	Overexpression of MSX2 generated immature MSCs, but with small molecules, mature MSCs were generated, and their trilineage potential was similar to that of BMMSCs	(138)
	EZH2 inhibitor was added to hESC medium, and cells were sorted	hESC	MSCs were generated within 7 days; no CD105 markers were used, and the generated MSCs had low adipogenic and chondrogenic potential but good osteogenic potential	(153)

**Table 3:** **Advantages and disadvantages of various PSC to MSC differentiation techniques.** Some differentiation techniques are superior that others in terms of length (time), economy (cost), the level of difficulty, and the yield (number of differentiated cells) of the technique

Techniques		Advantages	Disadvantages
Growth Factors		Easy and faster treatment, Multiple growth factor options available Efficient in 2D differentiation. Production of more homogeneous cell population	This method involves 2D differentiation thus does not mimic human in-vivo condition. Longer differentiation time that requires numerous sub-cultures. Requires enormous amount of growth factors
Embryoid Body		Mimics human physiological (in-vivo condition). Fast, more effective, and more efficient differentiation.	Possibility of necrotic zone formation. Production of heterogeneous cell population
Other techniques	OP9 monolayer cells	OP9 cells provide the necessary supplements, cytokines and growth factors for MSC differentiation. Results to production of cells with better trilineage differentiation.	Possibility of animal cells contamination. Longer differentiation time Additional sorting step is required
	Selective pressure	Homogeneous cell population. Animal free cells, Results to production of cells with better trilineage differentiation.	Longer time required, Negative effect of trypsin over cells, High batch-to-batch variations due to the complete dependency on cell passaging
	Genetic manipulations	Faster differentiation, Effective and efficient differentiation Results to production of cells with better trilineage differentiation.	Trained laboratory technicians required. Costly, Possibility of genetic mutations.
	Epigenetic inhibitor	Fast differentiation, good osteogenic potential	Results to production of cells with poor trilineage differentiation.

### Growth factor and small molecule-induced differentiation

The proliferation and differentiation of cells depend on their signaling pathways. These pathways involve intrinsic or extrinsic signaling molecules. Cells do not experience fate change without utilizing these signaling molecules, and same is the case for MSCs. A complex network of signaling pathways is involved in MSC proliferation, differentiation, and generation. However, limited MSC signaling pathways have been explored thus far. Recently, MSCs have been generated from PSCs by manipulating environmental and signaling cues during PSC culture.

BMP4 has been used to obtain MSCs from the mesendodermal and trophoblast lineages by varying FGF2 incorporation [138–140]. Similarly, iPSC to MSC differentiation can be induced through the ectodermal lineage route. Zhang et al. identified neural crest cells (NCCs) as a fast and efficient route for MSC generation through MSX2 overexpression [141, 142]. MSX2 has been found to be affected by Smad1/5/8, which is a main downstream molecule in the BMP4 pathway. Therefore, BMP4 can regulate MSX2 expression to differentiate iPSCs into NCCs or mesendodermal cells. Apart from growth factors, the small molecule SB431542 has been used to derive MSCs from iPSCs [143–145]. This molecule inhibits the phosphorylation of Smad2/3, which, in turn, inhibits TGF-β1 signaling in the ESCs that generate mesodermal progenitors (MP) [144].

Wnt and basic fibroblast growth factor (bFGF) have been observed to affect MSC generation. A few researchers have used CHIR, a GSK3β inhibitor, to induce MSC characteristics in iPSC-derived NCCs, while other researchers have used bFGF for similar purposes [138, 139, 142, 146]. Other growth factors, siRNA and different small molecules used for MSC generation are listed in Table 2.

### EB formation

Embryoid body (EB) formation changes the effect of a 2D environment on cell growth. EBs are 3D aggregates of PSCs cultured in suspension media. When left in suspension media or on non-adherent plates, ESCs or iPSCs accumulate and form aggregates that have spheroidal structures (143–145). A few EB aggregate formation methods are based on the forced self-aggregation, hanging drop, and AggreWell™ methods [147, 150]. EBs are widely used to provide a 3D platform for generating cells of different lineages [148, 149].

Interestingly, EB formation can be used to generate MSCs from PSCs. MSCs are generally obtained as an outgrowth from EBs because they have a fibroblast-like morphology and extend themselves on flat surfaces to ensure their growth and survival. EBs were first generated by culturing small clumps of ESCs in suspension culture for 10 days and then transferring them to gelatin-coated plates that generate MSC-like cells with increased expressions of the CD73 and CD105 markers [151–153]. Some of these cells were then cultured in 10% human platelet lysate under MSC culture conditions. After 35 days, the growth of fibroblast-like cells was observed. Various other methods have been developed, as summarized in Table 2.

### Other techniques

Apart from the above-mentioned techniques for MSC generation, there are other methods that do not fall into the categories discussed herein but are important to produce MSCs from PSCs. One such technique is the use of murine OP9 stromal cells for deriving MSCs. Barberi et al. cultured hESC over OP9 monolayer cells to induce mesenchymal differentiation [154]. ). In this direct co-culture system, the OP9 cells provide the necessary cytokines and growth factors to support the differentiation of hESC to MSC. After several days of co-culture, the cells were harvested and sorted for CD73 + surface marker. The CD73 + cells were then expanded in αMEM without mouse stromal cells.

Another method is the inclusion of a xeno-free environment. Karlsson et al. attempted to derive mesenchymal progenitor cells (MPCs) through the selective pressure technique. First, ESCs were cultured in high-glucose media containing bFGF for several days. The cells were then enzymatically dissociated into single cells and sub-cultured on a new gelatin-coated dish. This process of cell culture, cell dissociation, and passaging were repeated several times until homogeneous cell populations are achieved [155]. After several passages, these cells that no longer express any ESC surface marker are then characterized as MPCs.

Another method to generate MSCs involves directly transducing iPSCs with *Hox* genes that are upregulated in vascular wall MSCs (VWMSCs) [156]. Lentiviral transduction of these factors differentiates mouse iPSCs into VWMSCs [142].

Probably the most recent technology, epigenetics has also been used as a unique method for differentiating PSCs into MSCs. The enhancer of zeste homolog 2 (EZH2), an epigenetic regulator, was inhibited, and hESCs were differentiated into MSCs three times more efficiently than that in the case without such inhibition [157].

## Biomaterials affecting MSC development

Chemical cues play an interesting role in the differentiation of PSCs into MSCs and have been used widely. Mostly, growth factors, small molecules, and biomolecules such as RNA and DNA are used for this purpose, and they exhibit highly effective differentiation abilities. In spite of its great successes, this line of research has several limitations. For instance, the lack of biomimetic properties, unstable efficiency of growth factors, and tissue maturation are the drawbacks of utilizing only chemical cues to differentiate PSCs into MSCs. In addition to chemical cues, proper growth or maturation of cells can only be achieved through the regulation of their physical cues by using materials that can act as an ECM for cells. Biomaterials, which are common nowadays and have been utilized in many recent studies, successfully provide these physical cues. By using biomaterials, cell-cell and cell-matrix interactions are regulated efficiently. These biomaterials can also be conjugated with different growth factors (GF) which in turn stabilizes these GF and regulate their release into media for cellular consumption. In addition, cell adhesion, differentiation, and maturation can also be regulated by biomaterials [162]. It is important to note that the use of biomaterials to regulate cellular functioning varies depending on their properties, nature, formulation, and interactions with cells.

### Types of biomaterials

#### Natural biomaterials

Natural biomaterials are from natural sources, such as human, plant, and animal sources. Owing to their organic origin, natural biomaterials are biocompatible and biodegradable. In addition, they possess all the factors required for cell adhesion, proliferation, and differentiation, which aids cellular functioning [163]. Materials in this category include proteins, polysaccharides, proteoglycans, and glycosaminoglycans, and each of them has specific advantages and disadvantages in terms of cell function. These naturally derived biomaterials have been used to culture different types of cells and develop advanced medical products with increased cell implantation efficiencies [151].

##### Gelatin

Gelatin is one of the most widely used conventional ECM proteins for preparing biomaterials. It is normally obtained from the irreversible denaturation of collagen protein, and both materials exhibit similar molecular properties and functions in cell culture and tissue engineering. Gelatin has been demonstrated to be effective in the isolation and mass production of early-stage BMMSC [164]. Gelatin contains the integrin binding moiety: arginine–glycine–aspartic acid (RGD) sequence. The binding of the integrins present on the surface of BMMSCs to the RGD peptide of gelatin promotes better attachment of cells to the substrate. The enhanced attachment in turn increased proliferation of cells, and thus mass expansion of BMMSC. [165, 166].

##### Collagen

Collagen, the most common protein available in the human ECM, is a biomaterial that can promote the differentiation of MSCs into the osteogenic lineage [123]. Expansion on a collagen type I-coated plate is adequate to induce high levels of osteogenic differentiation. Pericellular collagen I facilitate MSC adhesion and differentiation into chondrocytes [167]. It is important to note that compared with gelatin, the highly organized structure of collagen is still superior in promoting cell adhesion and proliferation at any seeding density [168]. This indicates that the full version of the ECM protein is better in activating cellular processes. However, gelatin is way cheaper and readily available. Moreover, gelatin is highly soluble and modifiable which are essential characteristics for biomaterials. Because of these reasons, gelatin is utilized more, especially in routinary cell culture experiments.

##### Other Natural Biomaterials

Recently, silk fibroin scaffold has been used in stem cell engineering and has been found to be effective for repairing bone, cartilage, ligament, and skin [169, 170]. In addition, it has been especially helpful in achieving cell attachment and growth, but it performs better when conjugated with other materials. Chen et al. reported that the surface modification of silk fibroin with arginyl-glycyl-aspartic acid (RGD) peptide resulted in higher BM stomal cell attachment, cell density, and collagen matrix formation [171]. Likewise, there are other biomaterials, such as hyaluronic acid for alleviating osteoarthritis symptoms, fibrin as a sealant, and alginate for ESC-derived beta cell encapsulation [169].

In general, natural biomaterials contain the RGD binding moieties that aid in the attachment of cells to the culture plate. The initial attachment of cells to a substrate is an essential step in the differentiation of PSC into MSC. Stronger attachment would generally ensures better cellular responses such as proliferation and differentiation. In the case of PSC to MSC differentiation, it has been shown that PSC differentiating to MSC express high levels of integrins α5β1, a known protein that binds to integrin. Thus, the use of fibronectin coated dishes were found to enhance the isolation of PSC-derived MSC in-vitro [172].

Although natural biomaterials are effective and efficient in inducing cellular responses, their use in cell and tissue engineering is limited due to some limitations. First, natural materials may not be easily accessible in large quantities and thus are not preferred in upscaled or routinary experiments[173, 174]. Another reason is that they are normally extracted from different living organisms which is a labor-intensive process. Upon extraction, these materials need to be further purified and devoid of contamination. Because of these, natural biomaterials are expensive compared with synthetic materials. Finally, due to the complexity of these full-sized ECM proteins, tuning its mechanical strength for specific purposes is rather inefficient. Modifications to the base material is also often limited [175]. As a result, some scientist shifted towards the development and use of synthetic materials.

#### Synthetic biomaterials

Apart from natural polymers, synthetic polymers are promising as biomaterials. Synthetic biomaterials are artificially derived polymeric materials that are synthesized by combining small monomeric units, for example, polyethylene glycol (PEG), poly(glycolic acid) [PGA], poly(d,l-lactic acid) [PLA], poly(d,l-lactic acid-*co*-glycolic acid) (PLGA), poly(3-hydroxybutyrate), and poly(ε-caprolactone) (PCL). These monomers are polymerized to form stable structures that can alter cellular activity. Unlike natural biomaterials, synthetic biomaterials are devoid of batch-to-batch variation, mechanically stronger, more durable, and their structures can be modified easily to achieve various porosities and permeabilities [173]. Generally, these materials are unreactive to cells owing to the presence of hydrophilic groups on their surfaces. However, their interaction can be modified by conjugating them with growth factors, proteins, and small molecules to facilitate cell−material interactions. One of the most commonly used conjugates is the RGD peptide, which induces the attachment of cells onto materials owing to the inert binding capacity of integrin on cell surfaces and conjugation the RGD peptide conjugated to the material [176]. However, when selecting a biomaterial to obtain a particular cellular activity, its biocompatibility, biodegradability, and toxicity must be considered carefully.

##### Polyethylene Glycol

PEG is a polyether compound derived from petroleum and used for medical purposes. PEG is a non-ionic, biocompatible, hydrophilic compound that can be crosslinked using several methods to modify its physicochemical and biological properties. When iMSCs were cultured in PEG diacrylate conjugated with RGD peptide, they efficiently differentiated into valve interstitial cells [177]. Other modifications such as *N*-cadherin-loaded PEG dimethacrylate hydrogels and poly(*N-isopropyl acrylamide-co-)PEG* supports expansion and differentiation of mESC and hPSCs respectively [178, 179].

##### Polyvinyl alcohol (PVA)

Polyvinyl alcohol (PVA) is another hydrophilic polymer that is prepared through the hydrolysis of polyvinyl acetate or vinyl ester. It is a biocompatible and non-toxic material that requires surface modification for cell adhesion. In a study, PVA-*co*-itaconic acid hydrogel grafted with fibronectin and oligopeptide derived from the cell ECM led to stable expansion of hPSCs for several passages without any signs of differentiation [180, 181]. The stiffness of the hydrogel was maintained at 25.3 kPa throughout the PSC proliferation test. The stiffness of this range is found to be favorable for stem cell proliferation, but differentiation was observed in hydrogel with higher stiffness. Thus, to increase stiffness of the material, PVA is combined with PEG which helped in the differentiation of hESC toward mesoderm[182].

##### Polylactic acid (PLA)/ Poly(d,l-lactic acid-*co*-glycolic acid) (PLGA)

Polylactic acid (PLA) is a hydrophobic, biocompatible, tunable, and non-immunogenic polymer that is commonly used as a scaffold in cell culture [183]. It has been used for MSC cell culture and differentiation. This polymer, however, is normally found blended with other polymers to obtain materials of various strengths, degradation rates, and thermal stabilities.

For example, co-polymerization of PLA with glycolic acid leads to the formation of PLGA copolymer. This copolymer has enhanced material properties that are important for cell and tissue culture. The PGA component imparts degradable properties, whereas the PLA component provides improved cell adhesion, which makes PLGA extremely useful for regulating various cellular activities [184]. In one study, MSC cultured over a PLGA scaffold containing simvastatin led to bone growth when transplanted at a bone defect site [185]. Having the capability for growth factor attachment and to conjugate proteins and small molecules, PLGA has been utilized in differentiating PSCs into MSCs via the controlled release of growth factors specific for MSC differentiation. For instance, the encapsulation of miRNA extracted from active protein kinase A (PKA) expressing ESC in PLGA nanoparticles induces mesoderm differentiation in ESC that further differentiate into MSC [186]. Apart from the type of biomaterials, their design and interaction with cells regulate changes in cellular properties.

### Various biomaterial designs and platforms affect cell behavior

Apart from the nature of the starting materials, the spatio-physical presentation of these materials to cells is equally important for designing a biomaterial-based platform to achieve the differentiation of iPSCs to MSCs. The design of a biomaterial imparts different cell behaviors and, in turn, affects cellular processes such as proliferation, differentiation, and survival. Such biomaterial platforms include scaffolds, hydrogels, micro/nanoparticles, and fibers. Depending on the goal of the research, the biomaterial design should be selected carefully to obtain optimum results. For instance, hydrogels provide cells with a 3D microenvironment for survival and contain pores that affect the proliferation and differentiation abilities of cells [187]. Similarly, microparticles are helpful for forming spheroids and avoiding necrotic zone formation within these spheroids [188]. Other important stem cell regulations are discussed in the subsequent paragraphs.

#### Hydrogels

Hydrogels are 3D polymers composed of hydrophilic compounds, and for this reason, they have the capacity to retain water molecules and swell in response to such retention. These structures are not impermeable solids; instead, they are porous materials that allow water or any solvent to enter wihtin them and expand [189, 190]. Hydrogels are generally composed of polymers with hydroxyl, carboxyl, and amide groups for interaction, hydrophilicity, and gelation. Different hydrogel formation methods have been developed, and each method imparts different physical and chemical properties for various applications. In tissue engineering, hydrogels are primarily prepared from various types of natural (chitosan, collagen, and gelatin) and synthetic (methacrylate, polyvinyl, and polyethylene glycol) materials [191]. By altering these materials and varying their concentrations, researchers can produce different hydrogels with wide ranges of compressibility and elasticity. Hydrogels are known for their biocompatibility and biodegradability, and they demonstrably support cell attachment, drug and growth factor delivery, and cell encapsulation. For these reasons, they have been used to culture various cells, including MSCs and PSCs [192].

One advancement in hydrogel research is the development of composite hydrogels that allow for the formation of different microenvironments through “switching” of the active component. For instance, a hydrogel composed of alginate and collagen can be switched from a composite (alginate–collagen) to a pure collagen hydrogel through subsequent chelation and alginate washing. In this setup, the “switch” from composite to pure alginate induces the differentiation of PSCs into the meso- and ectodermal lineages [193]. Moreover, hydrogels can be incorporated with microparticles to create composite hydrogels. Dong et al. incorporated stromal-derived factor-1 (SDF-1) and kartogenin into modified chitosan-silk fibroin hydrogel and PLGA particles, respectively, to increase BMMSC proliferation and migration [194].

#### Porous and solid scaffolds

Scaffolds are 3D constructs that can support cellular processes such as attachment, proliferation, and growth. Although hydrogels can be classified as scaffolds, not all scaffolds are composed of hydrogels, and they can be prepared from various materials, including a wide range of metals, non-metals, fibers, composites, and even decellularized tissues. Similar to hydrogels, scaffolds provide a base structure for cellular attachment and growth. They are more solid and less porous compared to hydrogels [189].

Depending on the purpose, several scaffold preparation methods have been developed to obtain scaffolds of various shapes, compositions, and porosities. The most widely used method is particulate leaching, which involves the dissolution of particles pre-incorporated within the base biomaterial. Upon dissolution, the material retains its shape while leaving permeable “pores.” This method is widely used to obtain porous scaffolds, in addition to other methods such as solvent casting, gas foaming, emulsion templating, fiber formation, electrospinning, and 3D printing [189].

Various scaffolds have been used to regulate the fate of MSCs. For instance, BMMSC migration, adhesion, and osteogenic differentiation were enhanced by using a mesenchymal- and preosteoclast-protein-enriched dental bone matrix scaffold [195]. This scaffold contained proteins that facilitated the migration of cells toward the scaffold. In addition, the natural stiffness of the DBM matrix enhanced osteogenic differentiation. In another study, it was found that a patterned porous honey/silk fibroin scaffold prepared by means of soft lithography enhanced the proliferation of seeded MSCs [196]. A similar result was observed when MSCs were cultured in chitosan calcium polyphosphate and pigeonite scaffold [197].

A few of these scaffolds play an essential role in PSC proliferation and differentiation. A peptide polyvinyl butyral-based polymer scaffold synthesized by means of acrylic polymerization led to effective proliferation and differentiation of PSCs into cells of the mesodermal lineage, and therefore, it can potentially be useful for generating MSCs [198].

#### Microparticles/nanoparticles

Micro/nanoparticles are small spherical particles that can deliver growth factors or small molecules for cell growth or differentiation. One of the interesting roles of these particles is stabilizing the growth factors, which lose their efficiency in a free soluble state [199]. In cell and tissue culture, microparticles can be conjugated with the growth factors necessary for various cell functions such as proliferation and differentiation. Moreover, microparticles with varying properties such as stiffness, size, and sensitivity to certain stimuli, such as temperature, can be synthesized to control the release of conjugated growth factors. For instance, a thermo- responsive poly-N-isopropylacrylamide (PNIPAM) loaded with retinoic acid (RA) nanoparticles was used to induce the differentiation of iPSCs into neural cells [200]. The release of RA in these nanoparticles was achieved by changing the incubation temperature of the cells. Similarly, by considering the degradation properties of microparticles, the release of matrilin3 and TGF-β3 was controlled to achieve effective differentiation of MSCs into chondrocytes [201].

Moreover, microparticles can be used to stabilize certain biomolecules that are rather sensitive such as nucleic acids. In one study, the siRNA of chondrin was stabilized when loaded in crosslinked gelatin microparticles. The stabilized siRNA, in turn, facilitated efficient osteogenic differentiation of MSCs [202, 203]. Microparticles can also be used to provide greater surface area for cell attachment. In one study, functionalization of PLA microparticles with aminopropylmethacrylamide led to increased MSC attachment on its surface and produced larger cell aggregates compared to those produced on non-functionalized surfaces [204]. In another study, an approach to use RGD peptide on microparticles to achieve selective isolation of MSCs from spontaneously differentiated ESCs by using fibronectin was described [172].

#### Micro/Nanofibers

Another type of biomaterial designs are the micro/nanofibers. These are elongated and extended materials with varying lengths and diameters, and they are useful for creating fibrous scaffolds. Unlike porous scaffolds, fibrous scaffolds are primarily produced by spinning various materials by using methods such as electrospinning, wet spinning, biospinning, interfacial complexation, microfluidic spinning, and melt spinning (extrusion). These different spinning techniques allow for the fabrication of fibers having varying diameters, lengths, elasticities, and overall strengths. In this manner, different types of fibers can be generated for various tissue engineering applications [205].

Apart from the fabrication technique, the mechanical and biochemical properties of fibers are highly dependent on the type of polymer/material used. A few studies have suggested that the combination of two or more polymers can increase the mechanical stability or enhanced the biocompatibility of fibers [206]. In one study, a hybrid scaffold generated by coating knitted scaffolds with a thin film of poly (ε-caprolactone) (group I), poly (D, L-lactide-co-glycolide) nanofibers (group II), and type 1 collagen (group III) enhanced BM stromal cell attachment owing to the optimal mechanical strength of the hybrid scaffold [207].

The surface topography of the spun fibers can contribute to the overall cellular response and development. For instance, MSCs cultured on aligned electrospun poly-L-lactide-co-ε-caprolactone fibers exhibited cell proliferation, enhanced spindle morphology, and increased chondrogenic potential as those cultured on randomly oriented fibers [208]. In another study, the alignment of PCL nanofibers affects the differentiation potential of ESCs. Cells outgrow from the differentiated EBs developed along the aligned grooves of nanofibers formed neurites [209]. Indeed, fiber orientations allow cells to change their morphology. This phenomenon can be attributed to changes in the expression of the FAK molecule, actin, and microtubules, which are major regulators of cell attachment and morphology [210].

## Existing biomaterial-based systems for differentiation of PSC*s* into MSCs

The differentiation of PSCs into different cells mostly involves the use of a 2D culture supplied with culture medium and growth factors, which limits the effects of other variables that influence their proliferation and differentiation. The in-vivo cell microenvironment plays an important role in cell functioning. Cells possess different receptors that are activated after their interaction with the components present in these microenvironments. Nowadays, biomaterials are used to mimic the role of the ECMs present in the human body. This allows cells to express genes at higher levels than in cultures without biomaterials [211]. These materials can influence cellular behavior through their controlled degradation rates, cell surface-environment interactions, biomolecule release, and varying stiffness [211]. In particular, PSCs express ECM-associated genes, which affects their differentiation and proliferation [212]. To improve the efficiency of PSCs, processed biomaterials derived from natural sources, such as human and animal proteins, and biocompatible synthetic materials can be used. In addition, various biomaterial-based culture techniques, such as monolayer or 3D culture, have been proven to affect cellular functions.

### Natural biomaterials for differentiation of PSCs into MSCs

**Fig. 4:**
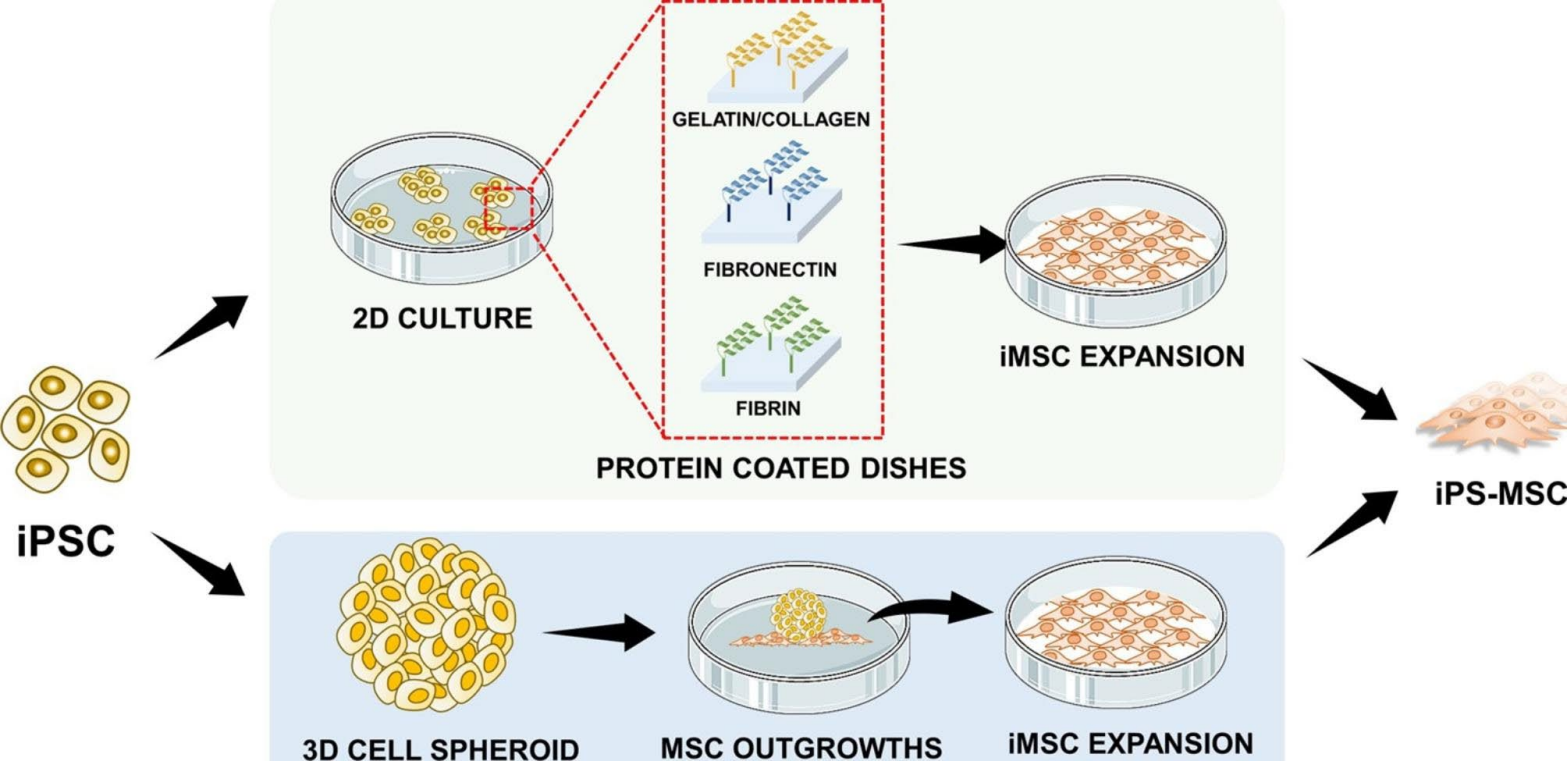
**Representative studies: Existing biomaterial-based technologies for the differentiation of PSC to MSC.** (a) Gelatin- and collagen-coated culture dishes as 2D systems for iPS-MSC generation by Obara. et al. [213]. (b) Fibronectin-coated dishes for efficient 2D isolation of iPSC-MSCs from spontaneously differentiated iPS cells by Cha et al. [172] (c) Embryonic bodies cultured in a gelatin-coated dish for generation of iPS-MSC outgrowths by Frobel et al. [216]. Images have been reused with permission

**Fig. 5:**
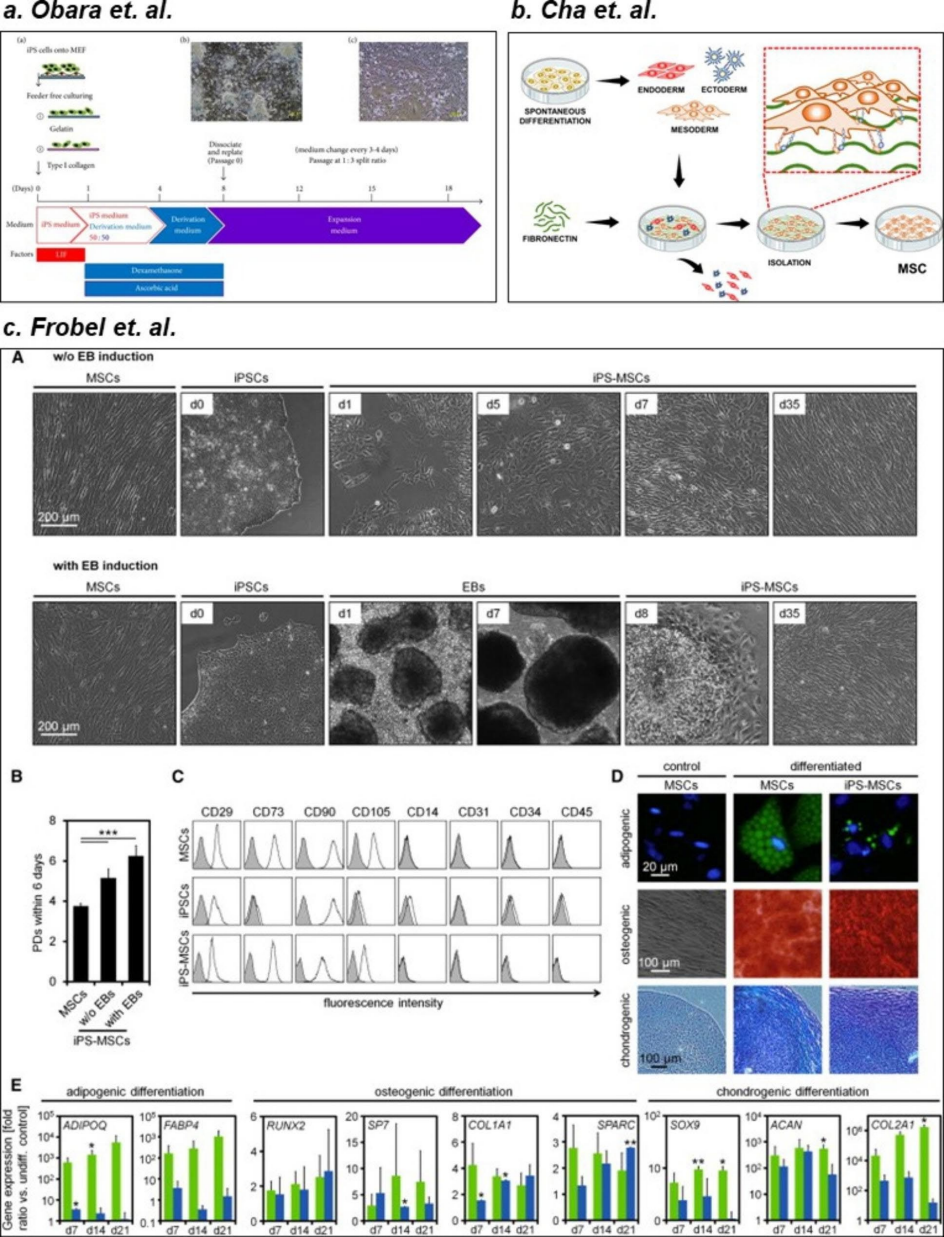
**Conventional 2D and 3D biomaterial-based differentiation of PSC to MSCs.** 2D systems generally involve repeated culturing of PSCs in dishes coated with ECM proteins such as gelatin, collagen, fibronectin, and fibrin. 3D systems, by contrast, involve the formation of embryonic bodies (cell spheroids), attachment on coated dishes, and collection of cell outgrowths (MSCs).

As described in the previous section, natural biomaterials affect MSC fate and are, therefore, great candidates for differentiating PSCs into MSCs. This is primarily attributed to the presence of cell-binding sites that support MSC induction and generation. For instance, gelatin and collagen are effective for deriving MSCs from murine iPSCs. Obara et al. cultured iPSCs on collagen- and gelatin-coated plates; after 8 days, fibroblast-like cells were generated, which were sub-cultured again on collagen- and gelatin-coated plates for further maturation, as depicted in Fig. 4a [213]. In another study, Liu et al. cultured PSCs, and their differentiation in collagen type I-coated culture plates increased the EMT of PSCs, which induced their differentiation into MSCs [214]. These researchers cultured dissociated single cells for MSC production because PSCs cultured in colonies did not exhibit the fibroblast-like morphology. In another study, iPSCs cultured on fibrin-coated plates differentiated into MSCs that exhibited enhanced fibronectin secretion and increased migratory activity. This result suggested that the attachment of cells in fibrin induced cytoskeletal changes in iPSCs, directing them toward MSC differentiation [215]. As for 3D systems, the conventional method involves formation of iPSC cell-spheroids called EBs and the attachment of these spheroids onto a surface coated with biomaterials such as gelatin, as described by Frobel, et al. Differentiated iPS-MSCs form cell outgrowths from these spheroids, and these outgrowths are collected for further characterization, as depicted in Fig. 4c [216]. Representative studies on a few of these biomaterial-based 2D and 3D systems are summarized and depicted in Fig. 5, and Fig. 4, respectively.

### Synthetic biomaterials for differentiation of PSC into MSCs

The properties of synthetic biomaterials have been shown to be effective for MSC derivation, provided that the material is modified to improve cell attachment for iPSC-derived MSC generation. Diaz et al. established a differentiation protocol for iPSC-derived MSCs by culturing iPSCs on poly(2-[methacryloyloxy] ethyl dimethyl-[3-sulfopropyl] ammonium hydroxide) (PMEDSAH)-coated culture plates; EBs were formed, which were then transferred onto gelatin-coated plates and differentiated into MSCs in an MSC medium [217]. These cells exhibited enhanced osteogenic properties, indicating changes in the metabolism of the iPSCs owing to the culturing material [218]. Although these materials were used for differentiation, their interactions with cells were not adequately illustrated.

Integrins are among the most studied molecules in terms of cell−ECM interactions. Integrin-associated signaling is a major mediator of biomaterial−cell interactions and is considered an important factor in the differentiation of PSCs [219]. For instance, ESCs cultured on polystyrene well plates engineered with conjugation of peptide ligands of integrin α5β1 and integrin α6β1 in mesodermal differentiation medium containing BMP4 expressed early mesodermal markers [220]. These cells exhibited remarkable differentiation potential toward the mesodermal lineage, indicating the possibility of producing MSC-like cells by using this method [221]. Some of these synthetic materials are mentioned in Table 4.

**Table 4:** **Various natural and synthetic materials for generation of MSCs from PSCs.** Several natural and synthetic materials have been utilized for efficient and effective differentiation of PSCs into MSCs. These materials strongly recapitulate the microenvironment in which MSCs reside in vivo, and therefore, provide a superior platform for MSC generation in in-vitro.

	Biomaterial	Method	Cells used	MSC properties	References
**Natural biomaterials**	**Gelatin and collagen**	One-step MSCs derivation method; gelatin and collagen are coated repeatedly for two passages over culture plates	miPSCs	CD73 is expressed; CD90 expression increases with passage, whereas CD105 expression decreases	(213)
	**Gelatin**	EB are formed for 7 days, and then they are cultured on 0.1% gelatin-coated plates for 2 weeks to attain confluency	hESCs	CD73 and Stro1 markers are expressed; MSCs are successfully differentiated into osteoblasts and adipocytes	(229, 230)
		iPSCs are cultured over 0.1% gelatin for 14 days in MSC medium	iPSCs	All major MSCs markers are expressed with stable passage capacity until 17 passages	(231)
		EBs formed are cultured in 1% gelatin-coated plates; some EBs attach and exhibit outgrowth; the unattached EBs are transferred to another 1% gelatin-coated plate in which MSC-like cells are formed in this culture.	hiPSCs	Both attached and transferred EBs form MSCs, which are termed as aiMSCs and tiMSCs, respectively. aiMSCs exhibit greater CD90 expression than tiMSCs. MSCs markers are expressed in the second passage	(232)
	**Collagen type I**	Collagen I is used to coat plates, PSCs are cultured over collagen, and repeated passaging is performed	iPSCs and hESCs	After the fourth passage, adult MSCs characteristics are gained; CD90, CD73, and CD105 are strongly expressed. Trilineage potential is observed, but with less adipogenic characteristics	(214)
	**Collagen type-IV**	EBs are cultured on collagen type IV-coated plate with growth factors to induce expressions of mesodermal and neuroepithelial cells, which are then converted into MSC-like cells in MSC medium for 1 month	hiPSCs	High expression of MSCs markers is observed; differences in paracrine factors can be seen	(233)
	**Fibrin**	Fibrin is used for differentiation, and both 2D and 3D environment are formed	hiPSCs	All MSCs markers are found; only adipogenic differentiation potential is found; 3D culture yields poorer results	(215)
	**Fibronectin**	ESC differentiates spontaneously and are then cultured in fibronectin-coated plates	hESCs	After 7 days, MSC markers are observed, isolation of MSCs from heterogeneous population is achieved; further passages generate mature MSCs with all surface markers and tri-lineage differentiation potential	(172)
**Synthetic biomaterials**	**PMEDSAH**	EBs formed from cells are cultured over PMEDSAH-coated plates and subsequently transferred to gelatin-coated plates for MSC generation	hiPSCs	CD90 markers are not observed; high adipogenic, chondrogenic, and osteogenic potentials are noted	(217)
	P**olypeptide conjugated gold-coated plates**	Integrin ligand-engineered plates are prepared; mesodermal differentiation medium supplemented with BMP4 is used	hESCs	After 48 h, mesodermal markers are observed: further downstream differentiation occurs	(220)

The use of different types of biomaterials is not the only factor that affects the differentiation of iPSCs. Another factor is the system in which the cells are cultured. 2D or 3D culture systems and how they are formed affect the cell proliferation and differentiation efficiencies of iPSCs. These factors regulate cell-cell and cell-matrix interactions, which further affect the cellular activity results.

### Two-dimensional (2D) systems for differentiation of PSCs into MSCs

2D cell culture is the conventional method, and it involves the growth and maintenance of cells on flat surfaces. This system provides cells with a substrate for attachment, which is necessary for growth and proliferation. The main drawback of using a 2D culture is that it does not fully recapitulate the physiological conditions of tissues in-vivo, wherein cells are arranged such that they can interact with the environment in 3D. Although 2D cell cultures may not fully represent tissues in vivo, much of the existing molecular biology and medical knowledge has been obtained using 2D cultures.

2D methods play a major role in the proliferation and differentiation of iPSCs into different cells. The use of biomaterials has improved cell maturity and efficacy. Both natural and synthetic materials, as well as hybrid forms, have been used to grow cells. In the 2D culture technique, biomaterials are generally used as a scaffold to provide iPSCs with the traction force needed to differentiate them into different lineages. iPSCs can differentiate into different lineages based on the stiffness, composition, and patterning of the biomaterial scaffold. Soft and stiff substrates support ectodermal and mesodermal lineages, respectively. This defines the effect of biomaterial stiffness on iPSC differentiation in 2D environments [222].

Similarly, a poly(dimethylsiloxane) (PDMS) scaffold with a stiffness of 100 Pa can be used to differentiate iPSCs into neural cells more efficiently than a substrate with a higher stiffness [223]. In another study, cells were induced to express the cardiac gene marker through electrical and mechanical signaling when a PLGA fiber scaffold coated with the conductive polymer polypyrrole was used as the substrate [224]. Similar culturing techniques have led to the efficient generation of MSCs from PSCs. Collagen-, gelatin-, fibrin-, and PMEDSAH-coated plates have been helpful for the conversion of PSCs to MSCs [213–215, 217]. In a study by Cha et al., ESCs cultured on fibronectin-coated plates in a spontaneous differentiation medium efficiently isolated and enhanced MSC-like cells among mixed populations of differentiated cells [172], as depicted in Fig. 4b. The MSCs expressed high levels of α5β1 integrin, a fibronectin receptor, which indicated that this method efficiently generated MSCs from PSCs. Similarly, it is important to identify the factors that affect cell physiology to produce MSC-like cells. These examples illustrate how 2D culturing methods can affect and even regulate the differentiation processes of cells.

### Three-dimensional (3D) systems for the differentiation of PSCs to MSCs

Culturing cells in a 3D environment is an efficient method for replicating the human physiological condition. Cell-cell and cell-ECM interactions are maintained and regulated from every plane in this culture system. There are two methods to create a 3D environment for iPSCs in in-vitro—one involves forming 3D cell aggregates called EBs and the other involves using biomaterials to encapsulate cells. For instance, neuron growth factor-conjugated alginate–chitosan–gelatin hydrogel allowed iPSCs to grow in a 3D environment, thereby enabling them to differentiate into neuron-like cells [225]. In a study by Zoldan et al., a 3D construct composed of a PLGA hydrogel with varying stiffness enhanced the differentiation efficiency by 50–80-fold [226]. Constructs with higher stiffness promoted the production of mesodermal lineage cells, whereas softer constructs promoted the production of endodermal lineage cells similar to the 2D methods but with higher expression [222]. Although this is an efficient method for iPSC differentiation, several results indicate that 3D methods generally produce smaller cell populations than 2D methods, which can be attributed to the formation of a necrotic zone at the center of the spheroid. This phenomenon has been observed in the 3D culture of fibrin-embedded iPSCs as well. The culture exhibited a heterogeneous population of MSCs with apoptotic clump formation and some fibroblastic colonies among the iPSC population, indicating the influence of limited nutrient supply at different locations in this 3D system [215]. However, this drawback can be resolved by incorporating gelatin microparticles inside the EBs to prevent the formation of necrotic cores [227]. Nevertheless, the biomaterial-based 3D culture of ESCs is a promising approach for inducing their differentiation into the mesodermal lineage [226].

A proper environment that supports efficient differentiation of iPSCs into MSCs must be considered. Little research has been conducted on the biomaterial-assisted differentiation of iPSCs into MSCs. However, the use of 0.1% gelatin in the differentiation of iPSCs into MSCs indicates that the cell surface-expressed αvβ3 and α5β1 integrins may be the primary factors driving this differentiation [166, 217]. Similarly, 3D environments composed of biomaterials can be developed. A 3D environment created from a PCL nanofiber scaffold with varying stiffness was used by Nam et al. to differentiate embryonic MPCs into different unipotent cells. This technique can be used to differentiate PSCs into MSCs because the stiffness of the scaffold supports mesodermal generation [219, 228].

## Future perspective on the biomaterial-based differentiation of iPSCs to MSCs

Recent advances in the differentiation of iPSCs to MSCs mainly include the regulation of molecular signaling pathways through the addition of growth factors or small molecules, whereas few studies have focused on the use of mechanical stimuli. Mechanical signals are provided by the cell’s surrounding ECM, and these signals, in turn, affect cell-cell and cell-ECM interactions to induce several molecular processes [234]. Although the exact mechanism by which the ECM regulates cell function has not been fully explored yet, it is hypothesized that mechanical signals affect integrin and laminin proteins expressed on the cell surface, which transfer the signal into the cell’s cytoskeleton. This activates the interaction between the Yes-associated protein (YAP) and the transcriptional coactivator with the PDZ-binding motif (TAZ), which eventually alters the cell’s behavior [235, 236].

Depending on the provided mechanical cue, several changes can be observed in cells subjected to mechanical stimuli. In the case of hPSCs, cells transitioned from the pluripotent state to the differentiated state when cultured over a switching hydrogel composed of alginate and collagen, with alginate degradation occurring before collagen degradation [193]. This change in the cell’s microenvironment initiated efficient differentiation of hPSCs into ectoderms within 3 days and into mesoderm and endoderm within 5 days. Similarly, modulation of the ECM stiffness can affect the differentiation of hPSCs. In one study, soft ECM (100 and 700 Pa) supported the neuronal differentiation of hPSCs, as indicated by increases in neurogenic and dopaminergic markers within 9 days [223]. Apart from stiffness, neuronal cells from iPSCs were obtained by varying the topographies of PCL through electrospinning [237]. Recent advances in biomaterials and methodologies include the introduction of various mechanical signals to aid the production of different cells from iPSCs [238]. Various methods that use these mechanical cues to generate cardiomyocytes from PSCs have been developed. Puig–Sanvicens et al. reported a biologically inspired self-assembling peptide hydrogel (RAD16-I) that could replace the supplement required for cardiac gene expression, which indicates that the mechanical cues derived from biomaterials can differentiate cells [239]. Similarly, the use of relatively stiffer substrates supports biomaterial in the differentiation process in forming mesoderm lineage cells [240, 241]. In a study by Quinton et al., a PDMS stiffness of 1.7 MPa led to greater mesodermal gene expression than a PDMS stiffness of 3 kPa, indicating the upregulation of mesodermal markers on relatively stiffer substrates [240]. These studies support the assertion that varying the stiffness and topology of biomaterials induces mechanical cues for the differentiation of hPSCs into mesodermal lineage cells [162]. This procedure can be further extended to produce MSCs from iPSCs because MSCs belong to the mesodermal lineage [221].

**Fig. 6:**
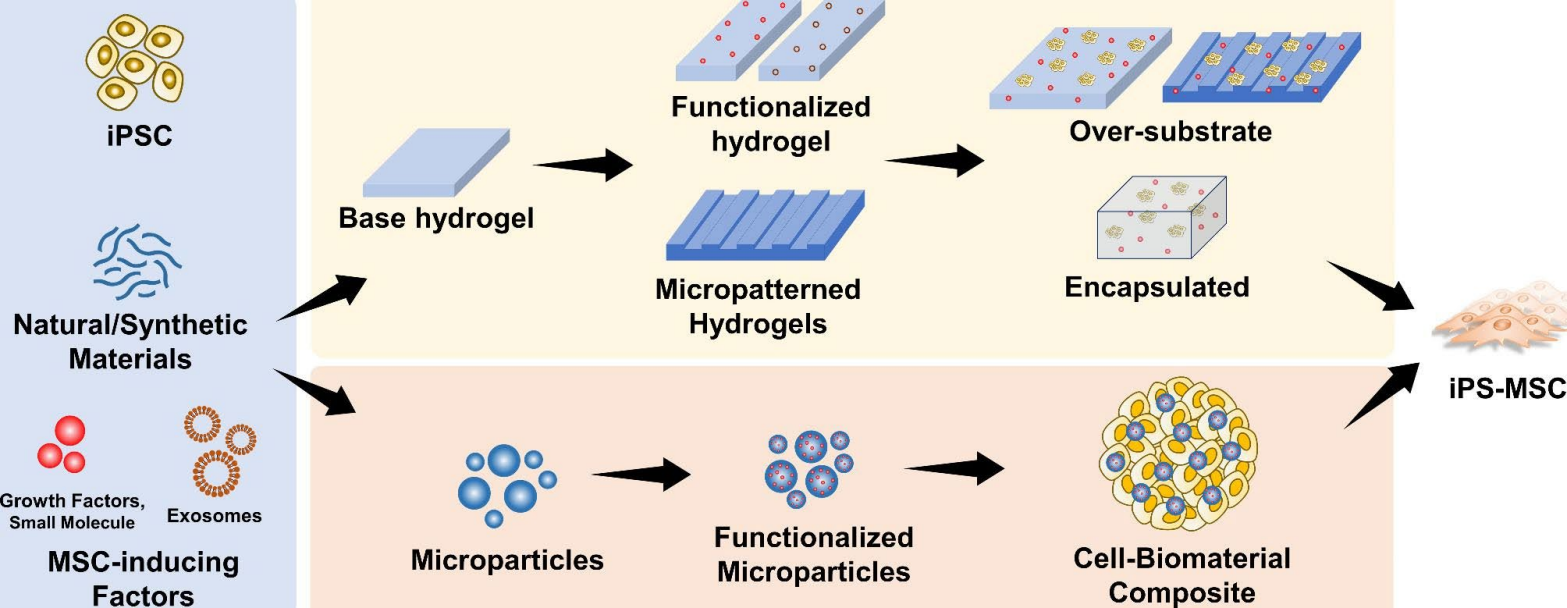
**Future prospects of biomaterial-based MSC generation from PSCs.** MSCs can be generated from iPSCs with the help of various biomimicking materials. Several natural and synthetic materials can induce the efficient growth and differentiation of MSCs. Functionalization of these materials through the conjugation of select MSC-inducing growth factors, exosomes, and modifications by varying stiffness and topology represent promising improvements to effectively and efficiently differentiate PSCs into MSCs.

Another potential method for MSC production is immobilization of protein on a biomaterial scaffold to initiate PSC differentiation. Immobilizing factors, such as BMP4 and SB431542 and inhibitors of nuclear factor-kappa B (IκB) kinase (IKK), on PDMS can induce the differentiation of iPSCs into MSCs [144, 159]. Although only a few studies have investigated the role of biomaterials and their impact on iPSC-derived MSC generation, similar studies have indicated the potential of using biomaterials to efficiently produce MSCs from iPSCs. However, proper selection of the base material, biomaterial system (2D versus 3D), correct stiffness, topology, and other MSC-inducing factors should be considered carefully to develop an ideal biomaterial-based system for producing MSCs from iPSCs [242]. The future scope for biomaterial-based MSC production is schematically depicted in Fig. 6.

## Conclusion

The use of MSCs for cell therapy and regenerative medicine has increased in recent years owing to the various properties of MSCs that are deemed important in cell and tissue regeneration. MSCs can differentiate into several cell types, such as osteocytes, chondrocytes, and adipocytes. Moreover, MSCs secrete various cytokines that promote the growth of neighboring cells or regulate immune responses. Owing to these properties, MSCs have been widely used in several clinical trials to treat tissue injuries, such as brain, heart, bone, and cartilage injuries. Their role in treating GVHDs has eased transplantation procedures. These results indicate the promising potential of the use of MSCs to achieve successful transplantations.

MSCs are adult stem cells that are present in almost all tissues of the body. However, variations in their proliferative capacity or differentiation potentials have been observed, and these characteristics are strongly dependent on the tissue and donor from which the cells are isolated. This heterogeneity is the major reason limiting the use of adult MSCs in cell therapy. These limitations can be resolved by deriving MSCs from PSCs. When PSCs are differentiated into MSCs through more defined and regulated procedures, an unlimited supply of clinically relevant MSCs can be obtained. Several strategies, such as growth factor induction, 3D culture, biomaterials, and epigenetics, have been studied. Each technique has unique advantages and disadvantages. However, given that the differentiation of iPSCs into MSCs can be induced via several techniques, it is suggested that a standardized protocol should be developed to realize better and more efficient production of clinically relevant MSCs for use in cell therapy and tissue regeneration.

## Electronic supplementary material

Below is the link to the electronic supplementary material.

Supplementary Material 1

Supplementary Material 2

## Data Availability

Not applicable.

## Abbreviations

AD: Adipose-derived
αMEM: Alpha-minimum essential medium
ADMSC: Adipose-derived mesenchymal stem cell
BDNF: Brain-derived neurotrophic factor
bFGF: Basic fibroblast growth factor
BM: Bone marrow
BMMSC: Bone marrow-derived mesenchymal stem cell
BMP: Bone morphogenic protein
CD: Cluster of differentiation
DC: Dendritic cell
DPMSC: Dental pulp-derived mesenchymal stem cell
EB: Embryoid body
ECM: Extracellular matrix
EMT: Epithelial−mesenchymal transition
ESC: Embryonic stem cell
EZH2: Enhancer of zeste homolog 2
FGF2: Fibroblast growth factor 2
GF: Growth factor
GVHD: Graft-versus-host disease
hESC: Human embryonic stem cell
HGF: Hepatocyte growth factor
hPSC: Human pluripotent stem cell
hiPSC: Human induced pluripotent stem cell
HLA: Human leukocyte antigen
HSC: Hematopoietic stem cell
iMSC: Induced mesenchymal stem cell
iPSC: Induced pluripotent stem cell
MHC: Major histocompatibility complex
MIP-1: Macrophage inflammatory protein-1
miPSC: Mouse induced pluripotent stem cell
MP: Mesodermal progenitor
MPC: Mesenchymal progenitor cell
MSC: Mesenchymal stem cell
MSX2: Muscle segment homeobox 2
NCC: Neural crest cell
NF-κB: Nuclear factor kappa B
PDMS: Poly(dimethylsiloxane)
PCL: Poly(ε-caprolactone)
PEG: Polyethylene glycol
PLA: Polylactic acid
PLGA: Poly(d,l-lactic acid-*co*-glycolic acid
PMEDSAH: Poly(2-[methacryloyloxy] ethyl dimethyl-[3-sulfopropyl] ammonium hydroxide)
PPARγ: Peroxisome proliferator-activated receptor gamma
PSC: Pluripotent stem cell
PVA: Polyvinyl alcohol
RA: Retinoic acid
RGD: Arginyl-glycyl-aspartic acid
RUNX-2: Runt-related transcription factor-2
SF: Synovial fluid
SFMSC: Synovial fluid-derived mesenchymal stem cell
SMMSC: Synovial membrane-derived mesenchymal stem cell
Stro-1: Stromal precursor antigen-1
TAZ: Transcriptional coactivator with PDZ-binding motif
TF: Transcription factor
TGF: Transforming growth factor
3D: Three-dimensional
TNFα: Tumor necrosis factor alpha
2D: Two-dimensional
UC: Umbilical cord
UCA: Umbilical cord artery
UCB: Umbilical cord blood
UCMSC: Umbilical cord-derived mesenchymal stem cell
UCV: Umbilical cord vein
VEGF: Vascular endothelial growth factor
VWMSC: Vascular wall mesenchymal stem cell
WJ: Wharton’s jelly
WJMSCs: Wharton’s jelly-derived mesenchymal stem cells
YAP: Yes-associated protein
