# Supplementary material for: Progress and emerging techniques for biomaterial-based derivation of mesenchymal stem cells (MSCs) from pluripotent stem cells (PSCs)
Source: Biomater Res. 2023 Apr 18;27:31. doi: 10.1186/s40824-023-00371-0 (PMC10114339; doi:10.1186/s40824-023-00371-0)
Supplement: Supplementary file 2 — Supplementary Material 2 [file 40824_2023_371_MOESM2_ESM.doc]

February 1, 2023

Prof. Byeongmoon Jeong

Editor-in-Chief

Biomaterials Research

Dear Prof. Jeong:

I wish to submit a review article for publication in the Biomaterials Research titled *“Progress and emerging techniques for biomaterial-based derivation of mesenchymal stem cells (MSCs) from pluripotent stem cells (PSCs)”.*

Cell-based therapies using MSCs have increased in the past decades. However current sources of MSCs show variabilities that arise from both donor– and tissue-specific differences. Moreover, individual cells from a single tissue source may exhibit heterogeneity indicated by the differences on their surface markers, which hinders the efficient use of these cells for therapeutic purposes. Because of this, researchers were prompted to generate methods to differentiate pluripotent stem cells such as embryonic stem cells (ESCs) and induced pluripotent stem cells (iPSCs) into clinically relevant MSCs.

In this review, the differences among MSCs derived from adult sources were compared and discussed. Moreover, the most recent technologies in the derivation of MSCs from iPSCs, with a focus on biomaterial-assisted methods in both two- and three-dimensional culture system are listed and elaborated. Finally, several opportunities to develop improved methods for the highly efficient biomaterial-assisted generation of MSCs toward advancing their various clinical applications are described.

This manuscript has not been published or presented elsewhere in part or in entirety and is not under consideration by another journal. We have read and understood your journal’s policies, and we believe that neither the manuscript nor the study violates any of these. There are no conflicts of interest to declare.

I would like to recommend potential reviewers below

1.	Ho-wook Jun (hwjun@uab.edu)

Professor, University of Alabama at Birmingham

Alabama, USA

2.	Seungpyo Hong (seungpyo.hong@wisc.edu)

Professor, School of Pharmacy

University of Wisconsin-Madison , USA

3.	Hsing-Wen Sung (hwsung@mx.nthu.edu.tw

Professor, Department of Chemical Engineering

National Tsing Hua University, Taiwan

4.	Won-Gun Koh (wongun@yonsei.ac.kr)

Professor, Yonsei University

Seoul, South Korea

5.	Kelvin Yeung ([wkkyeung@hku.hk](mailto:wkkyeung@hku.hk))

Professor, The University of Hong Kong Medical Center

Hong Kong, SAR

Thank you for your consideration. I look forward to hearing from you.

Sincerely,

Soo-Hong Lee

*soohong@dongguk.edu*

*Tel.: +82-031-961-5153*

*Fax: +82-31-961-5108*
